# Long-term colonization ecology of forest-dwelling species in a fragmented rural landscape – dispersal versus establishment

**DOI:** 10.1002/ece3.1163

**Published:** 2014-07-15

**Authors:** Kertu Lõhmus, Taavi Paal, Jaan Liira

**Affiliations:** 1 Institute of Ecology and Earth Sciences, University of TartuLai 40, 51005, Tartu, Estonia

**Keywords:** Conservation management, cultural heritage, ecological filters, forest landscape, functional traits, rural landscape, secondary succession, species traits

## Abstract

Species colonization in a new habitat patch is an efficiency indicator of biodiversity conservation. Colonization is a two-step process of dispersal and establishment, characterized by the compatibility of plant traits with landscape structure and habitat conditions. Therefore, ecological trait profiling of specialist species is initially required to estimate the relative importance of colonization filters. Old planted parks best satisfy the criteria of a newly created and structurally matured habitat for forest-dwelling plant species. We sampled species in 230 ancient deciduous forests (source habitat), 74 closed-canopy manor parks (target habitats), 151 linear wooded habitats (landscape corridors), and 97 open habitats (isolating matrix) in Estonia. We defined two species groups of interest: forest (107 species) and corridor specialists (53 species). An extra group of open habitat specialists was extracted for trait scaling. Differing from expectations, forest specialists have high plasticity in reproduction mechanisms: smaller seeds, larger dispersules, complementary selfing ability, and diversity of dispersal vectors. Forest specialists are shorter, less nutrient-demanding and mycorrhizal-dependent, stress-tolerant disturbance-sensitive competitors, while corridor specialists are large-seeded disturbance-tolerant competitors. About 40% of species from local species pools have immigrated into parks. The historic forest area, establishment-related traits, and stand quality enhance the colonization of forest specialists. The openness of landscape and mowing in the park facilitate corridor specialists. Species traits in parks vary between a forest and corridor specialist, except for earlier flowering and larger propagules. Forest species are not dispersal limited, but they continue to be limited by habitat properties even in the long term. Therefore, the shady parts of historic parks should be appreciated as important forest biodiversity-enhancing landscape structures. The habitat quality of secondary stands can be improved by nurturing a heterogeneous shrub and tree layer, and modest herb layer management.

## Introduction

Forest cover in Europe was severely reduced during medieval times (Grigg 1987), but during the last decades of the twentieth century, the forest area has increased mostly due to afforestation of former agricultural land (EEA 2009; Lindenmayer 2010). The annual global expansion of plantation forests is predicted to be more than 1% over the next 10 years (FAO 2011). Newly formed forest stands are, however, secondary forests by definition where the formation of suitable habitat conditions takes time and therefore do not always reconstitute the whole complex of ecosystem services provided by historically continuous forests (Brunet and Von Oheimb 1998; Lõhmus and Liira 2013). This maintains a tension between the newly created habitats with low levels of biodiversity but an increasing demand for timber products (Rudel et al. 2005) and the continuing degradation of forests with high biodiversity conservation value (Barbour et al. 2002; Foley et al. 2005). Therefore, nature conservation management cannot be restricted only to protected areas but must be integrated across the entire landscape, involving the enhancement of species migration between isolated historic and recently formed habitat fragments.

The colonization success of species is a function of time, as has been observed in recently afforested plantations (Harmer et al. 2001; Jacquemyn et al. 2003; Brunet et al. 2012). Much less is known about colonization drivers in secondary stands where habitat conditions have been stable for a longer period. In those stands, the importance of early successional processes has diminished and species have had time to positively react to changes (Herault et al. 2005). Most attention, regarding forest species colonization, has been paid to dispersal success and general characterization of forest plant-specific traits (Flinn and Vellend 2005; Hermy and Verheyen 2007). Forest plants are characterized as shade-tolerant species with dispersal limitation, which is mostly attributed to their relatively large seed mass and specialization to short-distance dispersal, such as myrmecochory (Brunet and Von Oheimb 1998; Takahashi and Kamitani 2004; Hermy and Verheyen 2007). However, the relative quantification of forest species' specificity in comparison with species of other ecological groups is rare. Therefore, it is not clear what defines forest specialist species, that is, their habitat needs or ability to react to landscape changes.

Colonization of species starts with dispersal, which is a combined function of landscape structure and species traits. This includes the availability of species in source habitats in the surrounding landscape (called local species pool – sensu Eriksson 1993; Pärtel et al. 1996), the landscape connectivity between the source and target habitat, and the dispersal properties of the species. The species pool availability is defined by the presence of historically continuous forest in the landscape and connectivity is supported by the current forest cover (Bellemare et al. 2002; Graae et al. 2004; Brunet et al. 2012). Dispersal across an unsuitable matrix (e.g., agricultural fields) to a target habitat is facilitated by dispersal corridors such as tree lines and hedgerows (Ehrlén and Eriksson 2000), as suggested by the patch-corridor-matrix theory (Opdam 1990; Forman 1995). However, the role of linear woody elements as corridor habitat for forest species is still being debated (Wehling and Diekmann 2009; Liira and Paal 2013).

The next stage of species colonization process is the species' establishment and persistence in the target habitat. Establishment success is determined by the interaction between plant traits and environmental conditions of a habitat (Honnay et al. 1999; Dupré and Ehrlén 2002; Normander et al. 2012). For instance, old-growth forests contain a higher diversity of microhabitats than young forest stands and therefore can harbor a higher diversity of forest-specific species (Brunet and Von Oheimb 1998; Rolstad et al. 2002; De Sanctis et al. 2010). However, newly formed forests or forest-like habitats with a complex stand structure may also provide more opportunities for immigrating species to become established (Bartemucci et al. 2006; Jamoneau et al. 2011; Liira et al. 2012). The importance of the structural quality of a newly formed habitat can, however, be overruled by the competition of generalist species, as forest specialist species are generally defined as poor competitors (Baeten et al. 2009; Brunet et al. 2011).

The relative importance of plant species traits and environmental factors in the process of colonization of newly formed habitats is still unclear. As most steps of the colonization process act at a relatively slow pace, newly formed stands with stable conditions should be addressed. The rural countryside includes planted habitats, which provide an opportunity to study the colonization process of slow-dispersing species. In eighteenth century Europe, the design of parks evolved from the French formal garden style toward the principles of English landscape gardens with dense tree stands planted around manor houses on traditional nonforest land, and these parks have, in the mean time, developed into forest-like habitats (Thacker 1981; Liira et al. 2012). Forest plantations and old parks have many common structural features, yet even a few differing characteristics can be critical in determining habitat quality (Lõhmus and Liira 2013). In forest plantations, tree growth and maximized timber production are core primary management goals, whereas supporting forest-dwelling biodiversity is a marginal secondary aim. In contrast, parks are planted and managed for aesthetical and recreational purposes, in which a diversity of microhabitats is already formed in early stages of stand development, and thus, shady parks provide a longer time window and more options for species establishment. However, the plantation management for biodiversity conservation is still based on quite subjective knowledge, and therefore, ecological profiling of forest species and the quantification of their ecology is still needed.

We address the complex of environmental factors and plant traits, which limit the colonization process of forest species in contemporary landscape, with emphasis that this is a long-term process and should be evaluated in late stages of community formation. Specifically, we targeted dispersal from historically continuous forests into newly formed forest-like habitats. Our first aim is to evaluate traits related to dispersal and establishment, which would distinguish forest confined species and corridor using species. To have adequate scaling of trait value positioning within potential ranges, we will use a comparison group of species from the matrix habitat. We compare species in three contrasting habitat types: (1) seed source habitats, represented by historically continuous nemoral or boreo-nemoral forest patches; (2) colonization target habitats, represented by old parks as forest-like habitats; (2) potential dispersal corridor habitats, represented by linear wooded habitats such as alleés, tree lines, and hedgerows. We scale the results using (4) surrounding open habitats represented by open nonagricultural habitats, such as grasslands and road verges. Our second aim is to estimate the roles of traits related to plant dispersal and establishment on colonization success in newly formed forest-like habitats, namely old parks. We will achieve this aim by creating a parsimonious model for predicting species colonization success in parks, including plant dispersal and establishment traits together with landscape and habitat metrics. The results would suggest not only how to improve forest conservation policy, but also how to evaluate the biological service of old-planted stands.

## Materials and Methods

### Study area

The study was conducted in central and southern Estonia (∼58°–59° N and 24°–27°30′ E) (Appendix S1). Cropland has, since the seventeenth century, been the predominant structure of landscapes surrounding rural Estonian manor houses (Liira et al. 2012) and since the late eighteenth century, parks around rural manor houses established on agricultural (Abner et al. 2007). The study area consisted of a mosaic of nemoral and boreal habitats, mainly on podzols, luvisols, and various gleysols with some areas on fluvisols; mean annual precipitation ranged from 600 to 700 mm, with an average summer temperature of 17°C in July, and an average winter temperature of −6°C in February.

### Sampling design

The sampling design comprised four habitat types: (1) forests; (2) rural parks; (3) corridors; and (4) open habitats. For rural park habitats, we selected closed-canopy remote park fragments dominated by deciduous old-growth trees. The average area of a fragment of homogeneous park stand was 3.4 ha. Both forest and corridor were selected within a 1 km radius around the park. Grasslands and road verges were sampled within a wider area around parks and forest as reference habitats to obtain a species list and a trait pool of open semi-natural habitats for scaling. We selected forest stands located on ancient forest land (i.e., continuously forested according to historical records) with an area of >0.9 ha and a forest stand age of >75 years. The stand structure, characterized by canopy closure and stand density, had to be comparable with adjacent forests. Suitable forests were preselected, based on various map data from the Estonian Land Board's Web Map Server (xgis.maaamet.ee), Estonian Forest Register (http://register.metsad.ee), and sources of Estonian Environmental Database (EELIS). A detailed comparison of stand structure between these parks and neighboring forests is described in the study of Lõhmus and Liira (2013). Major similarities between forest and park were light conditions and stand densities, whereas the main differences were in overstory compositions and understory management intensities.

We did not perform soil analyses as we sampled habitats on similar soil types and assumed that all target habitat types, that is, park, corridor, and grassland, have similarly been beneficially influenced by humans throughout history toward more nutrient-rich, optimized moisture regimes and physical structures (Honnay et al. 2002; Brunet et al. 2011). Instead, we limited our sampling to analogous soil types located on fresh soils of medium to high productivity, without estimation of current soil chemical status.

Corridor habitats were selected from linear wooded habitats such as hedgerows or tree lines and ranged from relatively young hedgerows to centuries old allées. However, most of these corridors are historically younger than forests, and therefore, we consider corridors as potential connecting habitats.

Altogether we sampled 230 forests, 74 parks, 151 corridors, and 97 grasslands.

### Data collection

Plant data collection was carried out from late May to early August 2008–2012, when both spring and summer plants were visible. In forest and park sites, we recorded all vascular plant species in a 30 m radius, sampling plot. In corridors, we sampled the area under the canopy's projection on the ground in a 30 m section that was at least 50 away from the forest or park. As grassland habitats were only used as a reference group to provide scaling estimates, we recorded all the observed herb layer species.

To evaluate the effect of habitat quality on species establishment in park habitats, we recorded stand structure characteristics and signs of various disturbances as indicators of habitat quality (Liira and Sepp 2009; Lõhmus and Liira 2013). We expected that stand structure determines various above-ground factors, particularly spatio-temporal light conditions, while management and disturbances determine conditions for establishment. We described the vertical canopy structure by visual estimation of canopy closure of the tree layer and the cover of foliar layers at three height intervals (1–4 m, 4–10 m, and >10 m) and described the composition of overstory and understory. We calculated a management index using signs of management such as mowing, cutting of trees and shrubs (detailed description of calculating management index in Liira and Sepp 2009; Liira et al. 2012). To estimate the historical and present-day landscape configuration around parks, we measured the proportional area of woodland and agricultural areas from historical maps (1890–1934) and present-day maps (2009) available at Estonian Land Board (xgis.maaamet.ee).

Data on plant traits were obtained from online databases and literature (Weiher and Keddy 1999; Pywell et al. 2003; Appendix S2). We collected data for (1) life-strategy traits including Ellenberg's indicator values (Ellenberg et al. 1991), hemeroby level (Jalas 1955; Sukopp 1969), and Grime's plant strategies (Grime 1979); (2) resource acquirement–related traits including average height, specific leaf area, growth form, leaf form, and the presence of petioles; and (3) dispersal-related traits, including flowering period, bright flowers, pollination vector, reproduction strategy, average seed mass, maximum dispersule mass, and dispersal vector. The study follows the plant nomenclature as found in Kukk (1999).

### Statistical analysis

Statistical analyses consist of two subsections: (1) the definition of species group of interest, and the comparison of trait patterns in that group with other species groups of reference; and (2) building a predictive model of colonization success of shade-tolerant species (forest and corridor dwellers) into new habitats (old parks).

We excluded infrequent species (<10 observations per species) and cultivars as we were interested in predicting the colonization success of common indigenous species. First, we analyzed the species affiliation with forest, park, corridor, and open habitats on the basis of occurrence patterns using Nonmetric Multidimensional Scaling (NMS) with Sørensen distance, random starting configuration, and 50 iterations with real data in PC-ORD v6.05 (McCune and Mefford 2011). The final two-axis solution had a stress value of 20.57 and instability <0.0001. We then used habitat scores from NMS to define three emergent groups of species: (1) forest specialists; (2) corridor specialists; and (3) open habitat specialists. A cut-off value between point clouds of habitats along the first axis was set so that equal proportion of sites would have been misclassified. That would also allow equal misclassification of species on the borderline between two neighboring habitat types. The NMS ordination included park habitats to illustrate the park's potential as a habitat of forest species, but park samples were not used to classify emergent groups of species.

To quantify the trait pattern differences between species groups and habitat types, we used general mixed-effect models in SAS v 9.2 (Littell et al. 1996). In these tests, we used the average value of each trait per emergent group in a site as a response variable and species group as a repeated fixed factor. As some habitat replicates were in relative close proximity to each other and thus cannot be considered as independent replicates in space, all habitats were nested within a local landscape window. The landscape window was defined as a 2-km buffer zone around a sample point (as a doubled landscape sampling radius around a park indicating the overlap between two neighboring park landscapes) and intersecting buffer zones were merged. We performed statistical comparison only between groups of forest and corridor specialists, as they were groups of interest, and only between closed-canopy habitat types: forest, park, and corridor. We used open habitat specialists only as an out-group for scaling general trends on graphs.

We built a generalized mixed-effect model using SAS v 9.2 (Littell et al. 1996) to explain the species colonization success from local species pool into the parks. We used species dispersal traits and niche-related traits, characteristics of habitat quality, and metrics of landscape structure as explanatory variables. Landscape window and species were included in the model as random factors. A local species pool was generated as a cumulative list of species for each 2-km landscape window around a particular park. We used a two-way stepwise selection of factors and Akaike information criterion (AIC) to build a model with a parsimonious set of explanatory variables.

## Results

**Figure 1:**
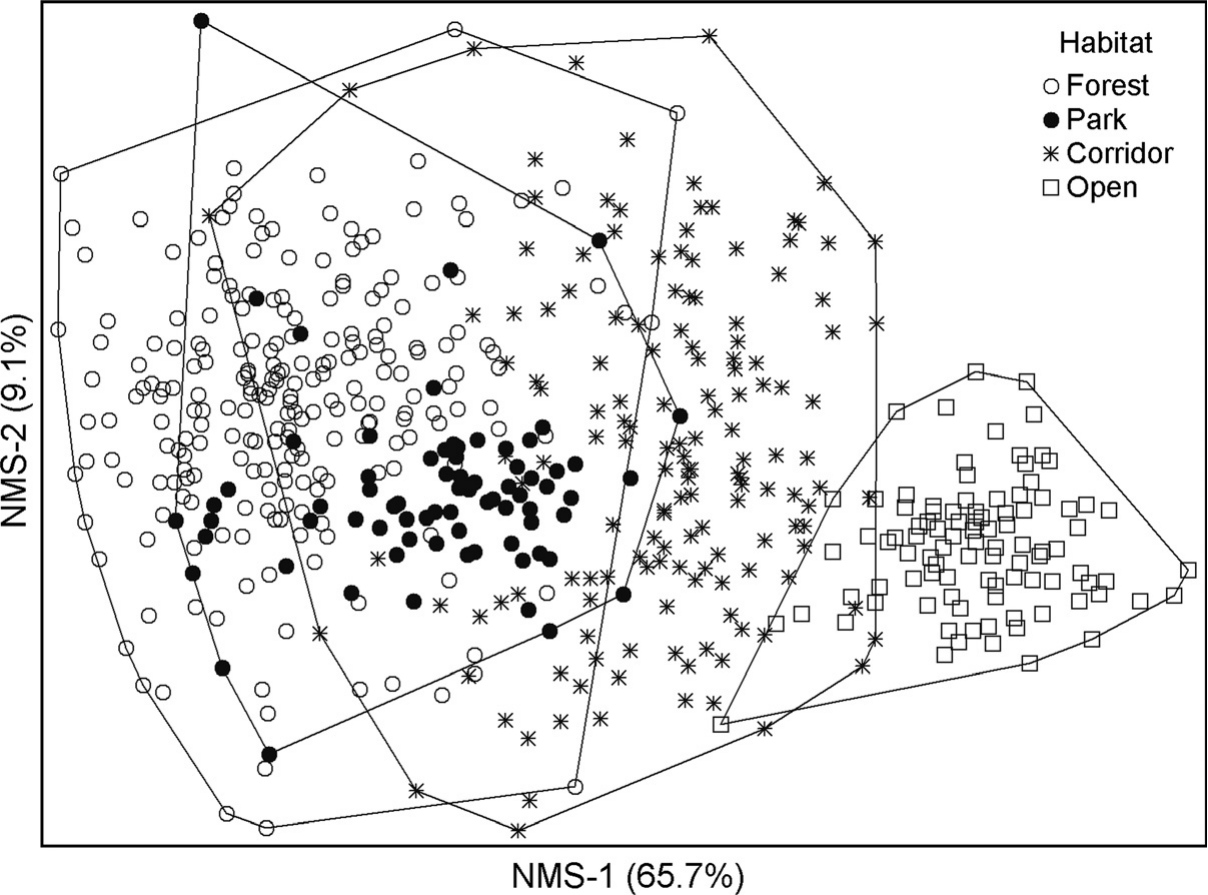
The Nonmetric Multidimensional Scaling ordination graph of sampling sites indicating a clear environmental gradient from closed habitats to open habitats, and the distinction of specialist groups for each habitat.

The first two axes of the NMS ordination explained 75% of the variation in the community data. Most of this variation was combined in the first axis (66%) and reflected a gradient from closed-canopy forest habitats to open grassland habitats, with parks and corridors in between (Fig. 1). The classification of species based on the NMS-1 scores resulted in 107 forest specialists, 53 corridor specialists, and 56 open habitat specialists. We used these emergent groups of species in all subsequent analyses.

Most trait patterns in parks showed intermediate traits values or values similar to the source habitat, and therefore, we will present a comparison between two extreme habitats. The summarized table for the models is presented in Appendix S3.

### Patterns in reproduction and dispersal traits

Forest specialists, on average, efficiently used both vegetative and generative reproduction (73% of species; Fig. 2.1), while corridor and open habitat specialists were equal in using generative reproduction (ca 47%; Fig. 2.2). Both seed- and dispersule-based dispersal were common among forest species (60%), while the dispersule-based type prevailed among corridor and open habitat specialists (80%; Fig. 2.3). Average seed mass of forest specialists was about half of that of corridor specialists, and this contrast did not deviate much between habitats (Fig. 2.4). Dispersule mass, however, did not differ between species groups, but only between habitat types. The dispersule mass was the largest in the parks and smallest in the corridors, but all these estimates were larger than those of open habitats (Fig. 2.5).

**Figure 2:**
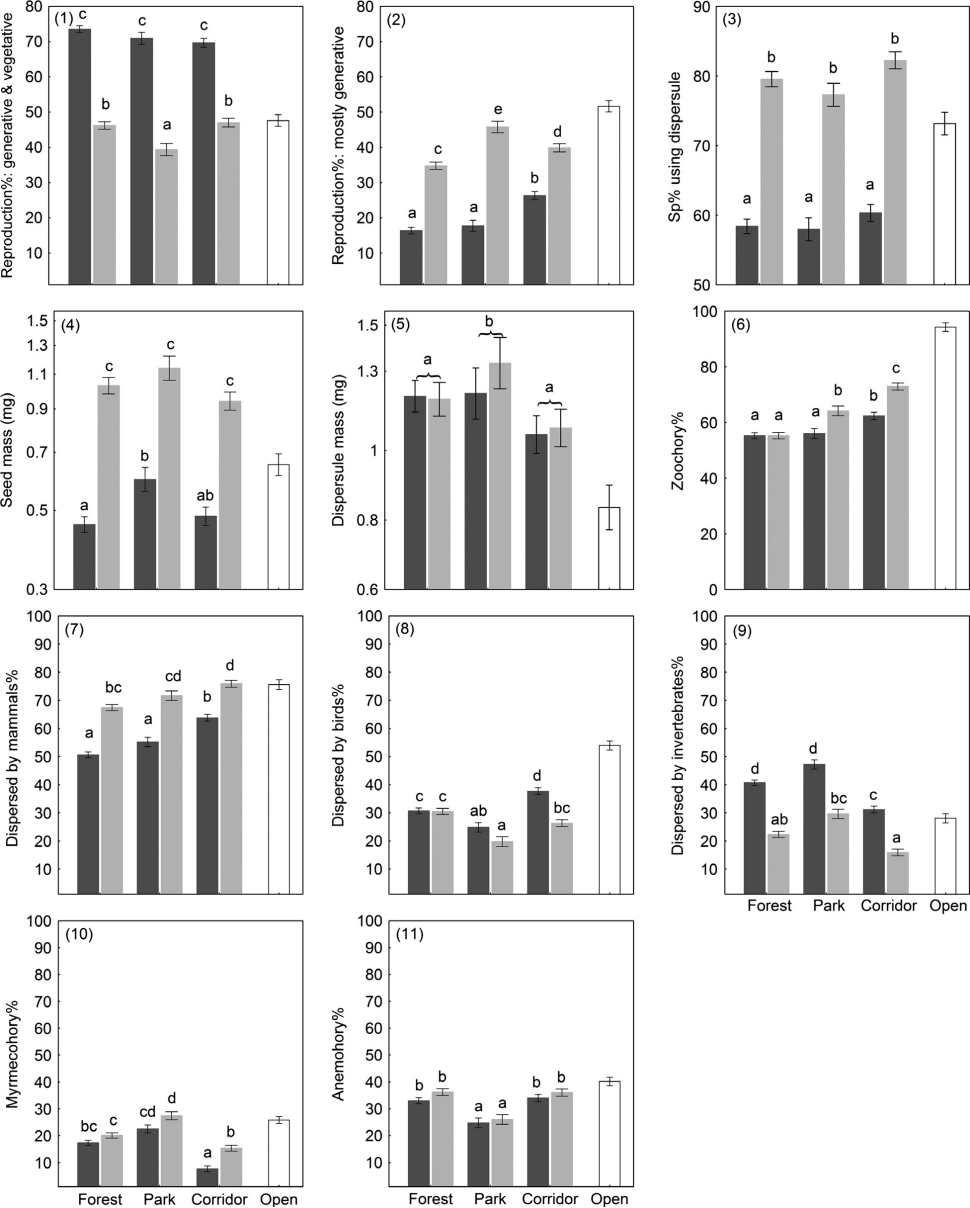
Dispersal trait patterns per species group across habitat types (means ± standard errors). Different letters within a figure indicate a significant pairwise difference (Tukey's post hoc test, *P *<* *0.05). Bar coloration represents species group (forest specialists = dark gray; corridor specialists = light gray; open habitat specialists = open bars).

Zoochory was the prevailing dispersal syndrome (>60%) in all three response groups, and its dominance over other types increased toward open habitats, particularly among corridor species (Fig. 2.6). Within zoochory, mammals were the most common vector, particularly in corridors and open habitats (75%; Fig. 2.7), while bird dispersal was half as common (Fig. 2.8). Dispersal by invertebrates was twice as important for forest specialists (40%) than for corridor specialists (12%; Fig. 2.9); but when only myrmecochory was considered then the contrast was not detectable (ca 15% for both species group; Fig. 2.10). The proportion of species using anemochory was analogous among forest and corridor specialists (ca 35%) in both habitats, while in parks, anemochory was less occasional (ca 25%; Fig. 2.11).

The proportion of species with bright-colored flowers was (about 10%) greater in both specialist groups inhabiting alternative habitat, that is, forest specialists in corridors and corridors specialists in forests and parks (Fig. 3.1). Analogous patterning was observed for the proportion of species with a biotic pollination strategy and inverse patterning for abiotic pollination (Fig. 3.2 and 3.3). All groups, particularly forest specialists, additionally had a high degree of ability for self-pollination (more than 60% of species had self–pollination ability; Fig. 3.4). Flowering of forest and corridors specialists occurred earlier in the season than open habitat specialists, but the flowering period of corridor specialists lasted about half a month longer than the flowering of forest specialists (Fig. 3.5).

**Figure 3:**
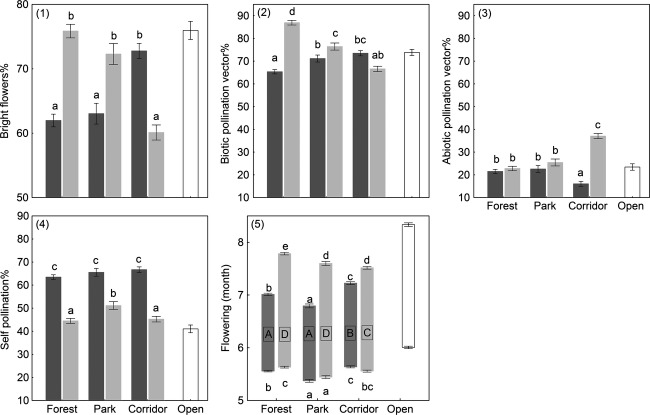
Flowering-related trait patterns per species group across habitat types (means ± standard errors). Different letters within a figure indicate a significant pairwise difference (Tukey's post hoc test, *P *<* *0.05). Bar coloration represents species group (forest specialists = dark gray; corridor specialists = light gray; open habitat specialists = open bars).

### Patterns of establishment traits

Forest specialists had lower Ellenberg's indicator values for light than corridor specialists (Fig. 4.1). Both specialist groups were represented by more light demanding species in corridors and this light requirement trend was supported by even higher light indicator values of open habitat species. Leaf and plant growth traits had coherent patterns. Specific leaf area (SLA) was relatively large for both specialist groups, only corridor specialists in corridors had smaller SLA, but still remarkably larger than for open habitat specialists (Fig. 4.2). Similarly, both forest and corridor specialists in forest habitats had a greater proportion of species (average of 60% of species) with leaf petioles than in corridor habitats. By contrast, a particularly low proportion of corridor specialists (41% of species) have leaf petiole in corridor habitats, which was comparable to open habitat specialists (Fig. 4.3).

**Figure 4:**
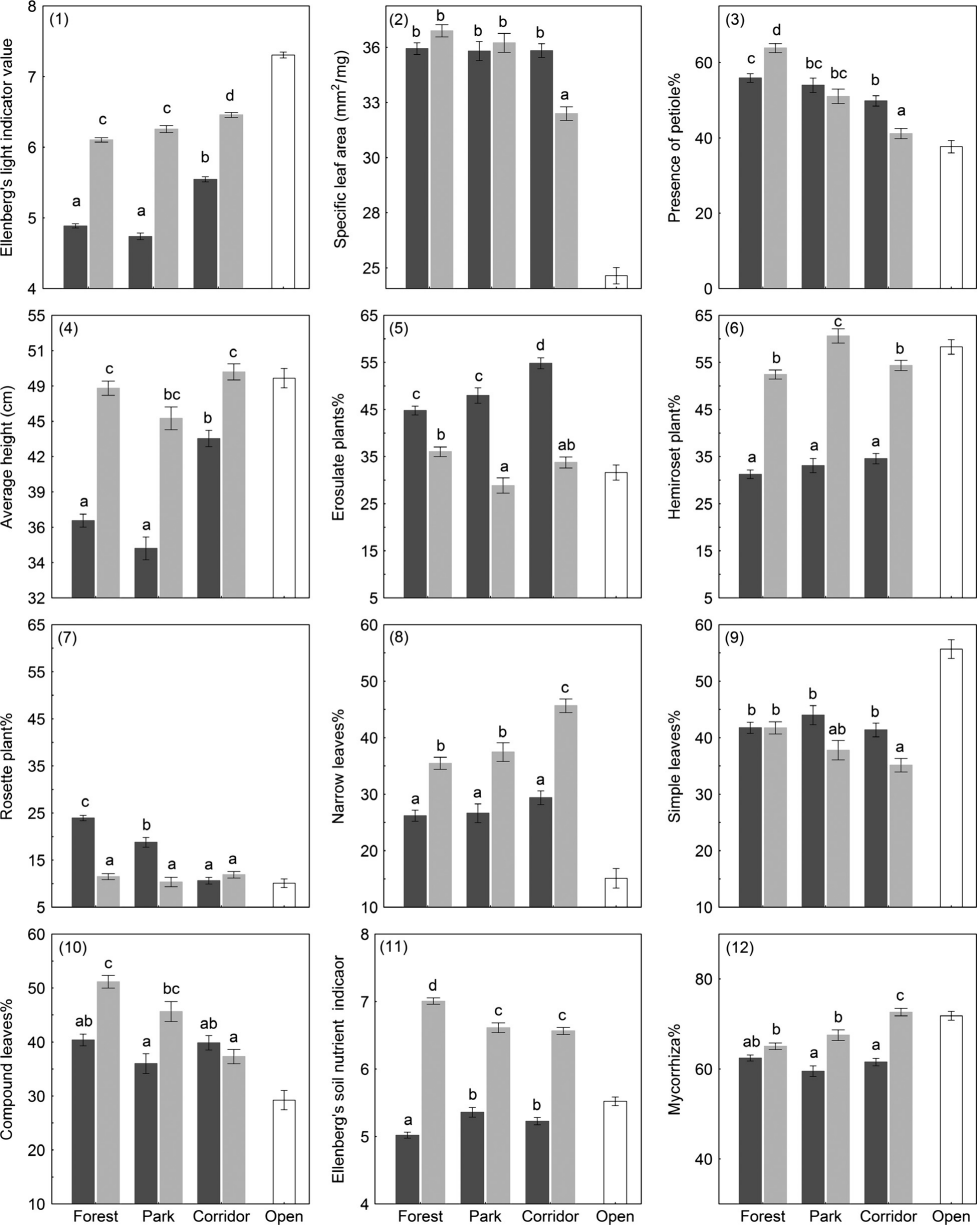
Resource acquirement trait patterns per species group across habitat types (means ± standard errors). Different letters within a figure indicate a significant pairwise difference (Tukey's post hoc test, *P *<* *0.05). Bar coloration represents species group (forest specialists = dark gray; corridor specialists = light gray; open habitat specialists = open bars).

Forest specialists had a shorter plant height in forest habitats than in corridor habitats, while corridor specialists were consistently tall in all habitats (Fig. 4.4). However, the erosulate growth form dominated among forest specialist (ca half of species) in forests and became especially prevailing in corridor habitats indicating the increased importance of vertical leaf positioning and competition between species (Fig. 4.5). In contrast, corridor and open habitat specialists were both dominated by species with a hemirosette growth form irrespective of habitat type (Fig. 4.6). The rosette growth form was mostly rare (ca 10%), except among forest specialists in forests (24%) and in parks (20%) (Fig. 4.7). The leaf shape type among forest specialists was constant in all three habitat types, while it varied between habitats in corridor specialists (Fig. 4.8–10) – the proportion of narrow leaves among corridor specialists decreased by about 10% from corridor to forest habitat and was replaced by simple and compound leaves. Open habitat specialists on the other hand were dominated by species with simple leaves.

Ellenberg's indicator value for soil productivity for forest specialists was low, as it was for open habitat species, while corridor specialists had much higher demands, and this contrast with forest specialists was strongest in forest habitats (Fig. 4.11). Mycorrhizal dependence as a special adaptation for acquisition of soil resources was more common among corridor species particularly in corridors (73%), which is comparable to open habitat specialists (Fig. 4.12).

Two types of strategy traits indicated the sensitivity of forest specialists. The level of hemeroby (the species tolerance to anthropogenic disturbances) was low for forest specialists in general, and particularly for those in forests and parks, while corridor and open habitat specialists had equally high disturbance tolerance (Fig. 5.1). Analogously, Grime's CSR-strategy indicators classified an average forest specialist's strategy as equally stress and competition tolerant (CS-strategy). The average corridor specialist had mostly the competitor strategy, but also partly the ruderal strategy (C- or CR-strategy) (Fig. 5.2–4), and the average open habitat specialist was defined as a CR-strategist.

**Figure 5:**
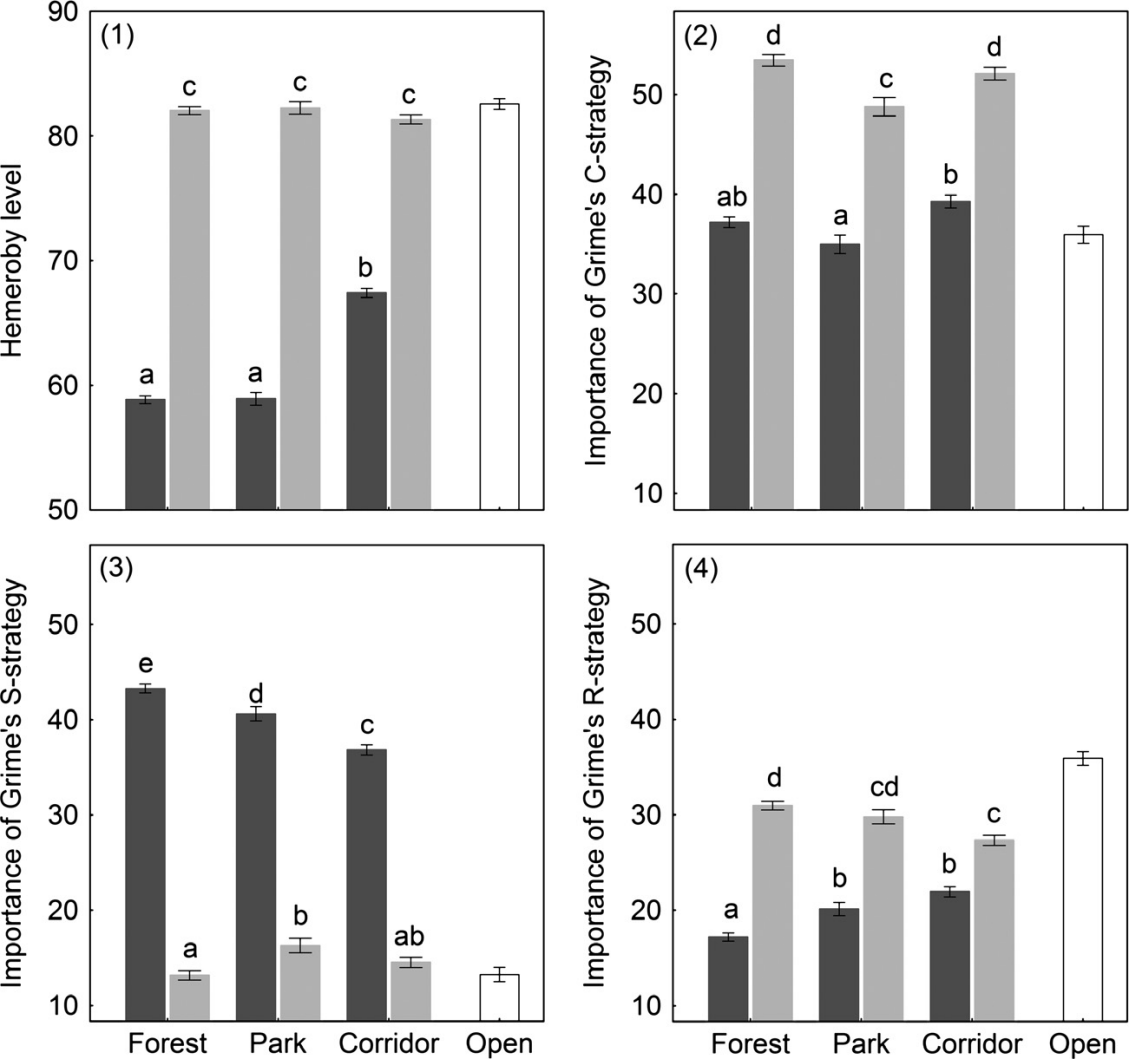
Life-strategy trait patterns per species group across habitat types (means ± standard errors). Different letters within a figure indicate a significant pairwise difference (Tukey's post hoc test, *P *<* *0.05). Bar coloration represents species group (forest specialists = dark gray; corridor specialists = light gray; open habitat specialists = open bars).

### Colonization prediction model

Herb layer richness of parks was on average 36 (SE ± 1.3) species, which consisted of forests specialists (63%) and corridor specialists (32%). At the landscape scale, the local species pool of forest specialists comprised an average of 40.2% (12.7–80.6%) of specialists in each particular park. Open land species amounted to an average 2 ± 0.3 species per park and can be neglected as a stochastic fraction of park biodiversity.

The results of the generalized linear mixed-effect model analysis showed that the colonization success of forest and corridor specialist species from local species pool can be explained by a combination of landscape configuration (ΔAIC = 21, compared with full model), habitat conditions (ΔAIC = 174), and plant traits related to life-history and resource use (ΔAIC = 39). Plant traits characterizing dispersal mode and propagule properties did not retain in the model. The landscape effect consisted of two factors: the proportion of historical forestland and the proportion of agricultural land in the area. The proportion of historical forests in the landscape predicted an increase in colonization probability of both forest and corridor species, while the proportion of agricultural land positively predicted the colonization of only corridor specialists (Table 1). The management intensity index of parks, as a generalized descriptor for anthropogenic disturbance activity, had a positive effect on the colonization success of both species groups. In addition to general stand management, the presence of mowing increased the colonization probability of corridor specialists. Understory abundance, that is, the foliage cover in the stratum of 1–4 m, had a positive effect on the colonization success of forest specialists and suppressed corridor specialists. Resource acquisition traits, which predicted species occurrences in parks, were shade tolerance (lower Ellenberg's indicator value for light) and earlier flowering time, and successful colonizers from the local species pool had a higher requirement for soil nutrients (Ellenberg's indicator for soil nutrients) and increased importance of Grimes' R-strategy.

**Table 1:** Results of the generalized linear mixed-effect model predicting the colonization success per species group (F = forest specialists; C = corridor specialists) to rural parks. The estimates indicate the direction and slope of the group variable. Landscape window and species were included as random factors. SE – standard error, *P* value *** < 0.0001, **< 0.01, *< 0.05, n.s – not significant.

Effect	*F* value	*P* value	Species group	Estimate	SE	*P* value
Species group (Sp.Gr.)	1.29	0.26	F	0.131	1.045	n.s
			C	−0.181	0.920	n.s
Landscape factors						
Historic forest cover (%)	7.71	0.006		0.014	0.005	*
Agricultural land cover (%)	3.77	0.052	F & C			
Sp.Gr. * Agricultural (%)	17.7	<0.001	F	−0.002	0.003	n.s
			C	0.015	0.005	**
Habitat properties						
Foliage layer 1–4 m cover (%)	0.74	0.39				
Sp.Gr. * Foliage 1–4 m (%)	72.0	<0.0001	F	0.011	0.003	***
			C	−0.016	0.003	***
Management	5.27	0.02	F & C	0.022	0.009	*
Mowing	13.9	0.0002				
Sp.Gr. * Mowing	24.8	<0.0001	F	0.125	0.154	n.s
			C	0.936	0.186	***
Species traits						
Flowering time	11.6	0.0007		−0.381	0.114	**
Ellenberg Indicator for light	7.35	0.007	F & C	−0.224	0.083	**
Ellenberg Indicator for soil nutrients	16.4	<0.0001	F & C	0.279	0.069	***
Grime's R-strategy	5.05	0.02	F & C	0.126	0.056	*

## Discussion

### Forest and corridor species

Biodiversity conservation can only be effective when the object of interest (i.e., an ecological group of species) and its limiting factors have been properly recognized. Therefore, the identification of a forest specialist species and its specific requirements for a habitat is an essential task. A forest specialist plant is traditionally characterized as a shade-tolerant species with poor competitive adaptations and limited dispersal capacity (Graae and Sunde 2000; Flinn and Vellend 2005; Brunet et al. 2012). The nearest ecological comparison group to forest specialists is the corridor specialists species group inhabiting woody corridors. Corridor specialists can be defined as shade tolerant generalists, which have a potential to be forest dwellers, but also use various alternative and perturbed shaded habitats in the landscape (Liira et al. 2012). Species of open habitats form a more distant group with more light-demanding species and higher competitive strategies. Hence, we expected the trait levels and environmental requirement values to reflect trait transition across emerged species groups, specifically from forest specialists to corridor specialists to open habitat specialists. Transitions across groups were indeed observed in several traits (e.g., light requirement, growth form), and not only across but also within species groups (e.g., several leaf properties, flowering characteristics and mycorrhizal dependence changed along the habitat gradient). However, such delineated comparisons revealed that only part of the traits and trait patterns agree with the traditional characterization of forest plants, probably because the comparison groups were rarely used to interpret the importance of each trait pattern.

Forest specialists were indeed best adapted to forest conditions as they are adapted to poorer light conditions and tolerated only low levels of anthropogenic disturbances. The adaptation to poorer soil conditions can be due to selective land transformation pressure by agriculture on productive soils. The shade tolerance of forest plants (Hermy et al. 1999; Brunet et al. 2011), but also corridor specialists, was supported by their leaf traits, flowering phenology and growth form. For instance, the prevalence of compound leaves with petiole or narrow elongated leaves indicates that forest species have adapted to avoid self-shading in already scarce light conditions (Falster and Westoby 2003). Additionally, forest species expressed fairly low competitive abilities as expected (Baeten et al. 2009; Brunet et al. 2011), but this feature is not unique to forest species, because they share it with open habitat species. Corridor species on the other hand showed a greater investment on competitive strategy as expected from their generalist nature (Roy and Blois 2006; Wehling and Diekmann 2009).

Dispersal-related traits on the other hand showed patterns that seemed to contradict earlier understandings (Matlack 1994; Ehrlén and Eriksson 2000; Verheyen et al. 2003b), for example, we found that forest specialists seem to be well adapted for dispersal. Forest species have smaller seeds than corridor species, which suggests that many seed-size drawn conclusions have been made based on the data of early successional forests. We suggest that the dispersule weight should be used to predict species dispersal ability (Liira and Paal 2013), because we also observed that dispersule weight was uniformly larger for forest and corridor species in comparison with open habitat specialists. Furthermore, our findings are contrary to former research that indicates forest specialists adaptations mostly for a short-distance dispersal (Couvreur et al. 2005; Hovstad et al. 2009; Peredo et al. 2013), one of which is myrmecochory (Hermy et al. 1999). In our results, the most common dispersal type in all species groups was zoochory, and its significance increased with open habitats among both forest and corridor specialists. Furthermore, wind dispersal, which is a long distance dispersal vector, was equally common in all species groups. Forest specialists are able to use long-distance dispersal vectors and thus dispersal cannot be a limiting factor. Additionally, forest species are shown to rely more on vegetative dispersal (Brunet et al. 2012), but we found that forest specialists are more flexible and use both vegetative and generative reproduction. At the same time, a combined reproduction strategy of generative and vegetative type was of less importance among other specialist groups, as they rely more on generative reproduction, indicating their more opportunistic ecology (MacArthur and Wilson 1967; Pianka 1970; Grime 1979).

An additional adaptation to forest conditions within the generative reproduction type of forest specialists is an early and short flowering period, and the potential deficit of pollinators in early season is compensated by self-pollination ability (Westoby 1998; Graae and Sunde 2000). Corridor species, similarly, had an early onset of flowering, but in contrast to forest species, they had a longer flowering period analogous to species of open habitats. Interestingly, in an alternative forest-like secondary habitat, such as a park, both forest and corridor specialists have even earlier onsets of flowering. They might indicate selective management pressure (Duflot et al. 2014) resulting from early summer mowing in parks to meet public expectations about park understory. The patterns of other flowering traits, such as a larger prevalence of species with bright-colored flowers and biotic pollination vector, indicate an evolutional reproduction advantage of insect-pollinated flowers in alternative habitats for specialist groups.

### What limits forest species? – Colonization model

As suggested by the emerged trait patterns of forest and corridor specialists, the colonization of a park stand was not limited by species dispersal traits. The prevalence of long-distance dispersal properties and the flexibility to use many reproductive types among forest specialists suggests that dispersal limitation has been an over-estimated factor, particularly when considering the colonization as a long-term process. The dispersal success of forest specialist species is correlated with landscape structure around forests (Verheyen et al. 2003a; Endels et al. 2007; Baeten et al. 2009) and specifically to historically continuous habitats (Graae et al. 2004; Ewers et al. 2013). We found that the importance of the long-term availability of seed-source habitats like historical forests in the landscape was important, but only for forest specialists. In contrast, shade-tolerant generalists, namely corridor specialists, benefited from the contemporary structure of the open landscape, where secondary habitats, including recently created corridors and woodland edges, might be important (Wehling and Diekmann 2009; Liira and Paal 2013).

We found that the colonization success of forest and corridor specialists was limited by the habitat quality of the target community (a park) and plant traits responding to some of the habitat quality defining conditions. As limited light availability and its variability are the base conditions for the formation of forest understory (Dupré and Ehrlén 2002; Bartemucci et al. 2006), we intentionally selected park stands on the basis of sufficient tree canopy closure, and therefore, overstory properties could not be the main factor in the colonization prediction model. Instead, smaller-scale factors, such as the cover of understory foliage layer (tree saplings and shrubs) was the best predictor of colonization success with specific effects on both response groups, as the shading effect by understory facilitated forest specialists and suppressed corridor specialists. This can be explained by the observed highest shade tolerance of forest specialist (Westoby 1998; Graae and Sunde 2000; Herault et al. 2005). Additionally, in parks where management was more sustainable, the establishment of both specialist groups was enhanced. We conclude that even though park management optimizes tree and shrub density for visitors, the average shade and intermediate disturbances seem to improve the overall environment in a stand toward an optimum condition for all shade-tolerant species (Von Oheimb and Härdtle 2009; De Keersmaeker et al. 2011; Liira and Paal 2013). However, some management treatments, such as intensive early summer mowing supported only corridor specialists, probably because corridor specialists with hemirosette growth form have higher mowing tolerance. This is reflected in the observed pattern of earlier flowering onset of forest species in park habitats in comparison with forest habitats. Another indicator group of forest specialists, intolerant of moving and management are ferns (McEvoy et al. 2006), which were largely absent in those habitats where intensive mowing was a component of the local management regime.

## Conclusion

The assembly formation in a new habitat is a large-scale process, where a long period of time integrates with the local species pool. Instead of using an overall richness estimate of all forest dwellers, the potential specificity of different specialist groups should always be taken into account. This study is one of a few about forest specialist plants, where the profound set of colonization factors was addressed simultaneously, and where the age of a planted forest was sufficiently old to avoid being a limiting factor. In the context of other specialist groups, a forest specialist plant can be characterized as a species with traits showing adaptation to tolerate intensive shade and lower soil nutrient levels, and intolerance to anthropogenic disturbances, but having a flexible reproductive and dispersal strategy, that is, they have an optimal trait composition to overcome habitat fragmentation. We found that corridor type habitats (hedges, alleys, step-stone patches) contain species with generalist type of traits and will little enhance forest biodiversity in open landscape.

Colonization success of forest specialists is determined by niche defining plant traits taking into account the habitat quality of the target habitat and the long-term availability of seed source habitats. Time is particularly important aspect in the process of planning new conservational hot-spots, where it is vital to consider not only the present-day status of the location (habitat patch) of interest and the simple number of species in it, but also to take into consideration its centuries old history and its surrounding landscape. Therefore, the primary task of conservation planning should be preserving the historical habitat patches and maintaining suitable conditions for habitat specialists in these fragments. Instead of the active creation of new habitats for forest-dwelling species, nature conservation should pay more attention to secondary habitats that have existed for some time and have acquired at least a fraction of the acceptable habitat quality; among them can be old parks. The role of old parks in rural landscape can be seen as analog of old traditionally managed grasslands and old small fields for grassland species.

The conservation of the biodiversity of old cultural habitats is easily achievable by focusing on the habitat quality requirements of the conservation value species or a more general group of habitat specialists. Many of these requirements do overlap with the close to nature management of public areas. For the enhancement of forest specialists, park management should promote a heterogeneous mid-story by mosaic understory landscaping, diversify seasonal variations in light conditions by supporting diverse overstory, and by mowing sufficiently “high” and late in the summer season. The resulting increase in biodiversity can deliver new attractions for public consumption; for instance, forest specialists can provide the visual and aromatic attractions of the mass flowering of spring ephemerals or the strictly visual patterns of structural leaf formations of ferns. These old parks are the first and sometimes the only easy-to-access educational spots about forest-like ecosystem in rural landscape.

## Supporting Information

Appendix S1 The map of study region with symbols denoting examined manor parks.

Appendix S2 List of databases and studies used for gathering information about plant traits.

Appendix S3 Results of the generalized linear mixed-effect models comparing the trait levels between species groups (forest specialists vs corridor specialists), habitat type (forest vs park vs corridor), considering the variation caused by local site and larger regional landscape window as random factor. *P* value *** < 0.0001, **<0.01,*<0.05, n.s – not significant.
