# Supplementary figures and images for: Long-term colonization ecology of forest-dwelling species in a fragmented rural landscape – dispersal versus establishment
Source: Ecol Evol. 2014 Jul 15;4(15):3113–26. doi: 10.1002/ece3.1163 (PMC4161184; doi:10.1002/ece3.1163)

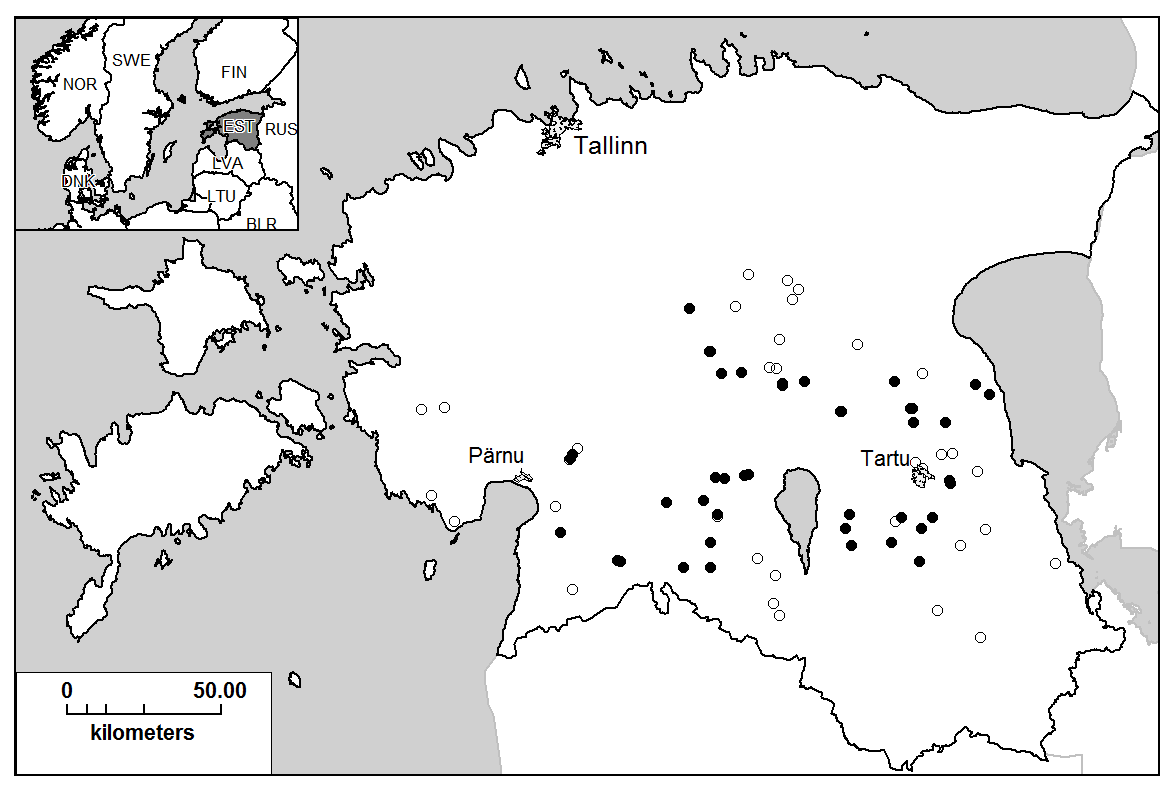

Supplement: Appendix S1 — The map of study region with symbols denoting examined manor parks. [file ece30004-3113-sd1.tif]
