# Supplementary material for: Long-term colonization ecology of forest-dwelling species in a fragmented rural landscape – dispersal versus establishment
Source: Ecol Evol. 2014 Jul 15;4(15):3113–26. doi: 10.1002/ece3.1163 (PMC4161184; doi:10.1002/ece3.1163)
Supplement: Appendix S2 — List of databases and studies used for gathering information about plant traits. [file ece30004-3113-sd2.docx]

**Appendix S2** List of databases and studies used for gathering information about plant traits.

Austrheim G, Evju M, Mysterud A (2005) Herb abundance and life-history traits in two contrasting alpine habitats in southern Norway. Plant Ecol 179:217-229.

Campbell JE, Gibson DJ (2001) The effect of seeds of exotic species transported via horse dung on vegetation along trail corridors. Plant Ecol 157:23-35.

Cosyns E, Claerbout S, Lamoot I, Hoffmann M (2005) Endozoochorous seed dispersal by cattle and horse in a spatially heterogeneous landscape. Plant Ecol 178:149-162.

Couvreur M, Vandenberghe B, Verheyen K, Hermy M (2004) An experimental assessment of seed adhesivity on animal furs. Seed Sci Res 14:147-159.

Davy AAJ (1980) Deschampsia caespitosa (L.) Beauv. J Ecol 68:1075-1096.

Dupré C, Ehrlén J (2002) Habitat configuration, species traits and plant distributions. J Ecol 90:796-805.

Dzwonko Z, Loster S (1992) Species richness and seed dispersal to secondary woods in southern Poland. J Biogeogr 19:195-204.

Eriksson O, Ehrlén J (1991) Phenological variation in fruit characteristics in vertebrate-dispersed plants. Oecologia 86:463-470.

Fischer SF, Poschlod P, Beinlich B (1996) Experimental studies on the dispersal of plants and animals on sheep in calcareous grasslands. J Appl Ecol 33:1206-1222.

Fitter AH, Peat HJ (1994) The ecological flora database. J Ecol 82:415-425.

Fröborg H (2001) Seed size and seedling emergence in 16 temperate forest herbs and one dwarf-shrub. Nord J Bot 21:373-384.

Gorb SN, Gorb EV (1995) Removal rates of seeds of five myrmecochorous plants by the ant Formica polyctena (Hymenoptera: Formicidae). Oikos 73:367-374.

Grime JP, Hodgson JG, Hunt R (1988) Comparative plant ecology: a functional approach to common British species. Allen and Unwin, London

Grime JP, Mason G, Curtis AV, Rodman J, Band SR, Mowforth MAG, Neal AM, Shaw S (1981) A comparative study of germination characteristics in a local flora. J Ecol 69:1017-1059.

Herault B, Honnay O (2005) The relative importance of local, regional and historical factors determining the distribution of plants in fragmented riverine forests: an emergent group approach. J Biogeogr 32:2069-2081.

Jongejans E, Telenius A (2001) Field experiments on seed dispersal by wind in ten umbelliferous species (Apiaceae). Plant Ecol 152:67-78.

Royal Botanic Gardens Kew (2008) Seed Information Database (SID). Version 7.1. Published on the Internet

<http://data.kew.org/sid/> (April 2013).

Kleyer, M., Bekker, R.M., Knevel, I.C., Bakker, J.P., Thompson, K., Sonnenschein, M., Poschlod, P., van Groenendael, J.M., Klimeš, L., Klimešova, J., Klotz, S., Rusch, G.M., Hermy, M., Adriaens, D., Boedeltje, G., Bossuyt, B., Dannemann, A., Endels, P., Götzenberger, L., Hodgson, J.G., Jackel, A.K., Kühn, I., Kunzmann, D., Ozinga, W.A., Römermann, C., Stadler, M., Schlegelmilch, J., Steendam, H.J., Tackenberg, O., Wilmann, B., Cornelissen, J.H.C., Eriksson, O., Garnier, E. & Peco, B. (2008) The LEDA Traitbase: a database of life-history traits of the Northwest European flora. Journal of Ecology, 96, 1266-1274.

Klotz, S., Kühn, I. & Durka, W. (2002) BiolFlor — Eine Datenbank zu biologisch-ökologischen Merkmalen der Gefäßpflanzen in Deutschland. Schriftenreihe für Vegetationskunde. Bundesamt für Naturschutz, Bonn.

Kukk, T (1999) Eesti taimestik. Teaduste Akadeemiea Kirjastus, Tartu, Estonia

Leht, M (2010) Eesti taimede määraja. Eesti Loodusfoto, Tartu, Estonia

Lindborg R (2007) Evaluating the distribution of plant life-history traits in relation to current and historical landscape configurations. J Ecol 95:555-564.

Pakeman RJ, Digneffe G, Small JL (2002) Ecological correlates of endozoochory by herbivores. Funct Ecol 16:296-304.

Poschlod, P., Kleyer, M., Jackel, A.K., Dannemann, A. & Tackenberg, O. (2003) BIOPOP - A database of plant traits and internet application for nature conservation. Folia Geobotanica, 38, 263-271.

Soukupová L (1992) Calamagrostis canescens: population biology of a clonal grass invading wetlands. Oikos 63:395-401.

Šerá B (2005) Diaspores – potential or real power of wild plants? Life cycle. Ekológia (Bratislava) 24:7-27.

Taylor K, Havill DC, Pearson J, Woodall J (2002) *Trientalis europaea* L. J Ecol 90:404-418.
