# Supplementary material for: Long-term colonization ecology of forest-dwelling species in a fragmented rural landscape – dispersal versus establishment
Source: Ecol Evol. 2014 Jul 15;4(15):3113–26. doi: 10.1002/ece3.1163 (PMC4161184; doi:10.1002/ece3.1163)
Supplement: Appendix S3 — Results of the generalized linear mixed-effect models comparing the trait levels between species groups (forest specialists vs corridor specialists), habitat type (forest vs park vs corridor), considering the variation caused by local site and larger regional landscape window as random factor. P value *** < 0.0001, **<0.01,*<0.05, n.s – not significant. [file ece30004-3113-sd3.docx]

**Appendix S3**Results of the generalized linear mixed-effect models comparing the trait levels between species groups (forest specialists vs corridor specialists), habitat type (forest vs park vs corridor), considering the variation caused by local site and larger regional landscape window as random factor. *P* value *** < 0.0001, **<0.01,*<0.05, n.s – not significant

|  |  | Fixed factors | | | Random factors | |
| --- | --- | --- | --- | --- | --- | --- |
| Trait | Units, scale / transformation | Species group | Habitat type | SpGroup*Habitat interaction | Landscape window | Site |
| **Resource acquirement** |  |  |  |  |  |  |
| Average height | [cm] / log | <0.0001 | <0.0001 | <0.0001 | ^n.s.^ | ^***^ |
| Specific leaf area | [mm^2^mg^-1^] / log | 0.022 | <0.0001 | <0.0001 | ^*^ | ^***^ |
| Rosette plant | 0/1 | <0.0001 | <0.0001 | <0.0001 | ^n.s.^ | ^***^ |
| Hemiroset plant | 0/1 | <0.0001 | <0.0001 | 0.009 | ^*^ | ^***^ |
| Erosulate plant | 0/1 | <0.0001 | <0.0001 | <0.0001 | ^n.s.^ | ^***^ |
| Narrow leaves | 0/1 | <0.0001 | <0.0001 | 0.004 | ^*^ | ^***^ |
| Simple leaves | 0/1 | <0.0001 | 0.001 | 0.001 | ^n.s.^ | ^***^ |
| Compound leaves | 0/1 | <0.0001 | <0.0001 | <0.0001 | ^*^ | ^***^ |
| Presence of petiole | 0/1 | 0.278 | <0.0001 | <0.0001 | ^*^ | ^***^ |
| Mycorrhiza | 0/1 | <0.0001 | <0.0001 | <0.0001 | ^n.s^ | ^***^ |
| **Dispersal** |  |  |  |  |  |  |
| Bright flowers | 0/1 | 0.001 | 0.027 | <0.0001 | ^*^ | ^***^ |
| Beginning of flowering | month | 0.204 | <0.0001 | <0.0001 | ^*^ | ^***^ |
| End of flowering | month | <0.0001 | <0.0001 | <0.0001 | ^*^ | ^***^ |
| Duration of flowering | months | <0.0001 | 0.320 | <0.0001 | ^*^ | ^***^ |
| Biotic pollination vector | 0/1 | <0.0001 | <0.0001 | <0.0001 | ^*^ | ^***^ |
| Abiotic pollination vector | 0/1 | <0.0001 | <0.0001 | <0.0001 | ^*^ | ^***^ |
| Self-pollination | 0/1 | <0.0001 | 0.003 | 0.055 | ^*^ | ^***^ |
| Reproduction mostly by seed | 0/1 | <0.0001 | <0.0001 | 0.000 | ^n.s.^ | ^***^ |
| Reproduction by seed and vegetatively | 0/1 | <0.0001 | 0.002 | 0.009 | ^n.s.^ | ^***^ |
| Reproduction mostly vegetatively | 0/1 | <0.0001 | <0.0001 | 0.000 | ^*^ | ^***^ |
| Seed mass | [mg]/ log | <0.0001 | 0.001 | 0.190 | ^n.s.^ | ^***^ |
| Dispersule mass | [mg]/ log | 0.488 | 0.016 | 0.730 | ^*^ | ^***^ |
| Zoochory | 0/1 | <0.0001 | <0.0001 | <0.0001 | ^*^ | ^***^ |
| Dispersal by mammals | 0/1 | <0.0001 | <0.0001 | 0.058 | ^*^ | ^***^ |
| Dispersal by birds | 0/1 | <0.0001 | <0.0001 | <0.0001 | ^*^ | ^***^ |
| Dispersal by invertebrates | 0/1 | <0.0001 | <0.0001 | 0.010 | ^*^ | ^***^ |
| Myrmecochory | 0/1 | <0.0001 | <0.0001 | 0.041 | ^*^ | ^***^ |
| Anemochory | 0/1 | 0.037 | <0.0001 | 0.728 | ^n.s.^ | ^***^ |
| Seed-Fruit dispersal | 0-seed/1-fruit | <0.0001 | 0.008 | 0.654 |  | ^***^ |
| **Life-strategy** |  |  |  |  |  |  |
| Ellenberg's light | 1-9 | <0.0001 | <0.0001 | <0.0001 | ^*^ | ^***^ |
| Ellenberg's soil productivity | 1-9 | <0.0001 | 0.063 | <0.0001 | ^*^ | ^***^ |
| Hemeroby level | 0...1 | <0.0001 | <0.0001 | <0.0001 | ^*^ | ^***^ |
| Grime's C-strategy (weight) | 0…100 | <0.0001 | <0.0001 | 0.012 | ^n.s.^ | ^***^ |
| Grime's S-strategy (weight) | 0…100 | <0.0001 | <0.0001 | <0.0001 | ^*^ | ^***^ |
| Grime's R-strategy (weight) | 0…100 | <0.0001 | 0.227 | <0.0001 | ^*^ | ^***^ |
